# Cytotoxic Prenylated Xanthones from the Pericarps of *Garcinia mangostana*

**DOI:** 10.3390/molecules19021820

**Published:** 2014-02-06

**Authors:** Zeng Xu, Lei Huang, Xiao-Hong Chen, Xiao-Feng Zhu, Xiao-Jun Qian, Gong-Kan Feng, Wen-Jian Lan, Hou-Jin Li

**Affiliations:** 1 School of Chemistry and Chemical Engineering, Sun Yat-Sen University, Guangzhou 510275, China; E-Mails: marinezengx@gmail.com (Z.X.); marinehuangl@gmail.com (L.H.); 2 Instrumental Analysis and Research Center, Sun Yat-Sen University, Guangzhou 510275, China; E-Mail: chxiaoh@mail.sysu.edu.cn; 3 State Key Laboratory of Oncology in South China, Cancer Center, Sun Yat-Sen University, Guangzhou 510060, China; E-Mails: zhuxfeng@mail.sysu.edu.cn (X.-F.Z.); marineqxj@gmail.com (X.-J.Q.) ; marinefenggk@gmail.com (G.-K.F.); 4 School of Pharmaceutical Sciences, Sun Yat-Sen University, Guangzhou 510006, China; 5 Guangdong Technology Research Center for Advanced Chinese Medicine, Guangzhou 510006, China

**Keywords:** *Garcinia mangostana*, xanthones, cytotoxic activities

## Abstract

Bioassay-guided fractionation of an ethanol extract of the pericarps of *Garcinia mangostana* led to the isolation of two new prenylated xanthones, named 1,3,7-trihydroxy-2-(3-methyl-2-butenyl)-8-(3-hydroxy-3-methylbutyl)-xanthone (**1**) and 1,3,8-trihydroxy-2-(3-methyl-2-butenyl)-4-(3-hydroxy-3-methylbutanoyl)-xanthone (**2**), together with the five known compounds garcinones C (**3**) and D (**4**), gartanin (**5**), xanthone I (**6**), and γ-mangostin (**7**). Their structures were elucidated primarily based on MS and NMR data. Compounds **1**–**7** showed significant cytotoxic activities against various human cancer cell lines.

## 1. Introduction

**Figure 1:**
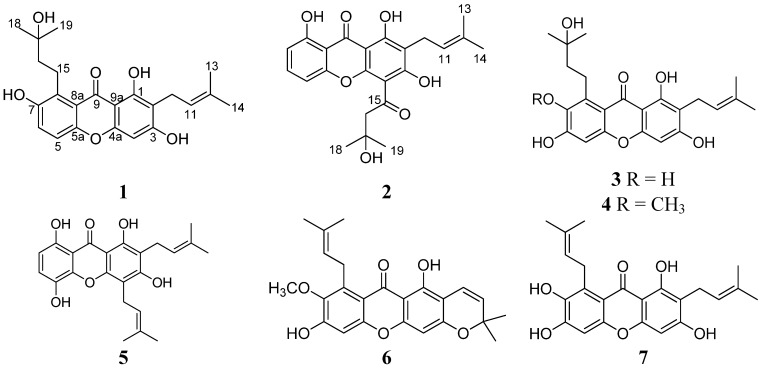
Chemical structures of compounds **1**–**7**.

*Garcinia mangostana*, commonly called mangosteen, is widely distributed in Southeast Asia. This tropical plant is famous for its nutritious and flavorful fruits. The pericarps have been used as folk medicine to treat the diarrhea, bladder infections, gonorrhea, and skin rashes for more than one hundred years. Previous chemical investigation of the whole fruits, trunks, branches, leaves, and pericarps afforded some oxygenated xanthones that have attracted much attention for their fascinating structures and a wide range of biological activities, such as anticancer, antifungal, antibacterial, antiviral, antioxidant, and antiplasmodial properties [1,2,3,4,5,6,7]. Recently, we carried out an HPLC-MS analysis of the ethanol extract of the pericarps, and found that the number of the HPLC peaks was much greater than the number of compounds isolated. Thus, the structures of some of the minor peaks are believed to still be unidentified. The semi-pure fractions were screened for cytotoxicity against the cancer cell lines CNE1 and CNE2, and showed the significant inhibitory activities, with IC_50_ values of less than 20 μg/mL. These findings aroused our great interest in further investigating these chemical constituents and their pharmacological activities. The ethanol extract of the pericarps was subjected to silica gel column chromatography followed by repeated HPLC to afford two new prenylated xanthone derivatives, 1,3,7-trihydroxy-2-(3-methyl-2-butenyl)-8-(3-hydroxy-3-methylbutyl)-xanthone (**1**) and 1,3,8-trihydroxy-2-(3-methyl-2-butenyl)-4-(3-hydroxy-3-methylbutanoyl)-xanthone (**2**), together with the five known compounds garcinones C (**3**) and D (**4**), gartanin (**5**), xanthone I (**6**), and γ-mangostin (**7**) (Figure 1). Compounds **1**–**7** showed significant cytotoxic activities against various human cancer cell lines. Herein, we describe the isolation, structure elucidation, and biological evaluation of these compounds.

## 2. Results and Discussion

**Figure 2:**
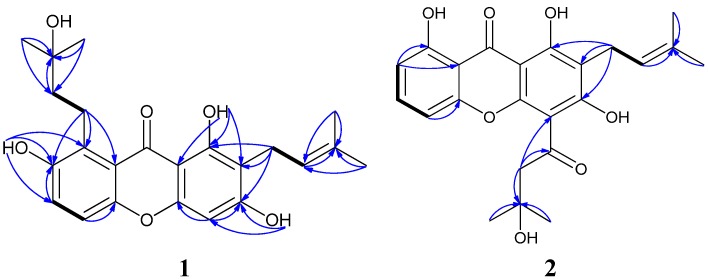
^1^H-^1^H COSY (bold line) and main HMBC (arrow) correlations of compounds **1** and **2**.

Compound **1** was obtained as a yellow powder with the molecular formula C_23_H_26_O_6_ and eleven degrees of unsaturation as established by its HREIMS peak at *m/z* 398.1725 [M]^+.^. The UV spectrum showed the characteristic absorption bands of a xanthone skeleton at λ_max_ 350, 287, and 247 nm. The ^13^C-NMR data and DEPT experiments displayed four methyls, three methylenes, four methines, and twelve quarternary carbons (Table 1). One carbonyl group at *δ*_C_ 184.3 and twelve aromatic carbons at *δ*_C_ 163.7, 161.9, 156.3, 152.5, 152.4, 131.1, 124.5, 119.6, 116.8, 111.1, 104.3 and 93.3 constituted the xanthone skeleton. In the ^1^H-^1^H COSY spectrum, the allyl proton at H-11 (*δ*_H_ 5.29, t, *J* = 7.2 Hz, 1H) displaying cross peaks with H-10 (*δ*_H_ 3.37, d, *J* = 7.2 Hz, 2H), H-13 (*δ*_H_ 1.79, s), and H-14 (*δ*_H_ 1.65, s), combined with the HMBC correlations between H-11, H-13, H-14 and C-12 (*δ*_C_ 131.6 (C)) (Figure 2) revealed that a prenylated 3-methyl-2-butenyl side chain in this molecule. ^1^H-^1^H COSY correlations of H-5 (*δ*_H_ 7.23, d, *J* = 9.2 Hz)/H-6 (*δ*_H_ 7.33, d, *J* = 9.2 Hz), and H-15 (*δ*_H_ 3.49, t, *J* = 7.6 Hz, 2H)/H-16 (*δ*_H_ 1.84, t, *J* = 7.6 Hz, 2H), revealed two partial structures –CHCH– and –CH_2_CH_2_–. The quarternary carbon at *δ*_C_ 71.1 (C-17) connected to two methyl groups (*δ*_C_ 29.8, C-18 and C-19), the hydroxyl group (*δ*_H_ 3.81, OH-18), and the above partial structure –CH_2_CH_2_–, and formed the other prenylated 3-hydroxy-3-methylbutyl side chain. Three phenolic hydroxyl groups at *δ*_H_ 13.69 (s, OH-1), 9.68 (s, OH-3) and 8.72 (s, OH-7), were placed at C-1, C-3, and C-7, respectively, based on the HMBC correlations from OH-1 to C-1, C-2 and C-9a, from OH-3 to C-2, C-3 and C-4, from OH-7 to C-6, C-7 and C-8. The hydroxyl group at *δ*_H_ 13.69 (s, OH-1) formed an intramolecular hydrogen bond with the carbonyl group at *δ*_C_ 184.3 (C-9). The HMBC correlations between H-10 and C-1, C-2 and C-3 indicated that the prenylated 3-methyl-2-butenyl side chain was connected to C-2, moreover, the correlations between H-15 and C-7, C-8 and C-8a confirmed the other 3-hydroxy-3-methylbutyl side chain was connected to C-8. Therefore, compound **1** was elucidated as 1,3,7-trihydroxy-2-(3-methyl-2-butenyl)-8-(3-hydroxy-3-methylbutyl)-xanthone.

Compound **2**, was obtained as a yellow amorphous solid. The molecular formula was determined to be C_23_H_24_O_7 _by analysis of the HREIMS peak at *m/z* 412.1517 [M]^+^ and its ^13^C-NMR data (Table 1). The ^13^C-NMR data and DEPT experiment displayed four methyls, two methylenes, four methines, and thirteen quarternary carbons. The cross-peaks of H-5 (*δ*_H_ 7.70, d, *J* = 8.0 Hz)/H-6 (*δ*_H_ 7.37, dd, *J* = 8.0, 7.6 Hz), H-6/H-7 (*δ*_H_ 7.44, d, *J* = 7.6 Hz) in ^1^H-^1^H COSY showed that three aromatic hydrogens formed a –CHCHCH– partial structure in this molecule (Figure 2). The HMBC correlations of H-16 (*δ*_H_ 3.67, s, 2H)/C-15 (*δ*_C_ 206.1), H-16/C-17 (*δ*_C_ 72.0), H-18 (*δ*_H_ 1.45, s)/C-17, and H-19 (*δ*_H_ 1.45, s)/C-17 indicated a 3-hydroxy-3-methylbutanoyl partial structure. The other 3-methyl-2-butenyl side chain was identical to that of compound **1**. The location of the side chains on the xanthone core were assigned at C-2 and C-4 by interpretation of the HMBC correlations of H-10/C-1, H-10/C-2, H-10/C-3, and H-16/C-4. Three phenolic hydroxyl groups at *δ*_H_ 14.33, 14.40 and 15.11 were located at C-1, C-3, and C-8, and they formed intramolecular hydrogen bonds with the carbonyl groups. Based on the analysis above, the structure of compound **2** was assigned as 1,3,8-trihydroxy-2-(3-methyl-2-butenyl)-4-(3-hydroxy-3-methylbutanoyl)-xanthone.

The known compounds were identified as garcinones C [8] and D [9] (**3** and **4**), gartanin [10,11] (**5**), xanthone I [12] (**6**), and γ-mangostin [13] (**7**), respectively, by comparison of their physical and spectroscopic data with those reported previously.

**Table 1:** ^1^H- and ^13^C- NMR Data of compounds **1** and **2** at 400 and 100 MHz, resp., in acetone-*d_6_*. *δ* in ppm, *J* in Hz.

Position	1			2	
	*δ*_H_	*δ*_C_		*δ*_H_	*δ*_C_
1		161.9			165.9
2		111.1			111.8
3		163.7			170.3
4	6.42 s	93.3			105.0
4a		156.3			158.3
5	7.23 d (9.2)	116.8		7.70 d (8.0)	115.9
5a		152.4			145.7
6	7.33 d (9.2)	124.5		7.37 dd (8.0, 7.6)	126.2
7		152.5		7.44 d (7.6)	121.9
8		131.1			147.4
8a		119.6			122.1
9		184.3			181.9
9a		104.3			103.0
10	3.37 d (7.2)	22.1		3.36 d (7.2)	21.6
11	5.29 t (7.2)	123.6		5.26 t (7.2)	122.3
12		131.6			132.5
13	1.79 s	18.1		1.80 s	18.0
14	1.65 s	26.0		1.66 s	25.9
15	3.49 t (7.6)	22.6			206.1
16	1.84 t (7.6)	44.5		3.67 s	55.3
17		71.1			72.0
18	1.30 s	29.8		1.45 s	30.4
19	1.30 s	29.8		1.45 s	30.4
OH-1	13.69 s			14.33 s *^a^*	
OH-3	9.68 s			14.40 s *^a^*	
OH-7	8.72 s				
OH-8				15.12 s *^a^*	
OH-18	3.81 s			2.81 s	

*^a^* These data may be interchanged.

The human nasopharyngeal carcinoma cell lines CNE1, CNE2, SUNE1 and HONE1, human lung cancer cell lines A549 and GLC82, human breast cancer cell line MCF-7, and human hepatic cancer cell line Bel-7402 were used to examine the cytotoxic activities of compounds **1**–**7** *in vitro*. Hirsutanol A was used as positive control. As can be seen in Table 2, compounds **1**–**7** showed significant cytotoxic activities against these human cancer cell lines. By comparison of their potencies on the tested cancer cell lines, compounds **1** and **2** are similar to compounds **3** and **7**, and greater than **4**–**6** and hirsutanol A.

**Table 2:** Cytotoxic activities of **1**–**7**, and hirsutanol A *in vitro*. IC_50_ values in µM *^a^*.

Cancer cell Lines	1	2	3	4	5	6	7	Hirsutanol A
Human nasopharyngeal carcinoma cell line CNE1	1.43	2.09	0.76	16.28	6.46	15.58	1.85	10.06
Human nasopharyngeal carcinoma cell line CNE2	0.73	1.21	0.54	9.04	4.51	9.77	1.81	12.70
Human nasopharyngeal carcinoma cell line SUNE1	2.23	2.76	2.30	24.06	8.78	11.36	4.41	3.53
Human nasopharyngeal carcinoma cell line HONE1	–	–	1.18	16.72	7.40	11.17	2.78	17.48
Human lung cancer cell line A549	–	–	1.97	20.17	–	15.92	3.79	12.05
Human lung cancer cell line GLC82	–	–	–	15.38	5.52	16.97	3.46	10.11
Human breast cancer cell line MCF-7	–	–	2.85	19.30	8.26	10.07	5.27	10.35
Human hepatic cancer cell line Bel–7402	–	–	3.32	16.73	7.78	11.38	3.66	24.80

*^a^* – = not screened.

## 3. Experimental

### 3.1. General Procedures

Optical rotations were measured on a Schmidt and Haensch polartronic HNQW5 optical rotation spectrometer (SCHMIDT + HAENSCH GmbH & Co., Berlin, Germany). IR spectra were obtained using a PerkinElmer Frontier FT-IR spectrophotometer (PerkinElmer Inc., Waltham, MA, USA) UV spectra were recorded on a Shimadzu UV-Vis-NIR spectrophotometer (Shimadzu Corporation, Nakagyo-ku, Kyoto, Japan). 1D and 2D NMR spectra were recorded on a Bruker Avance II 400 spectrometer (Bruker BioSpin AG, Industriestrasse 26, Fällanden, Switzerland). The chemical shifts are relative to the residual solvent signals (acetone-*d*_6_: *δ*_H_ 2.05 and *δ*_C_ 29.92; DMSO-*d*_6_: *δ*_H_ 2.50 and *δ*_C_ 39.51). Mass spectra were obtained on a Thermo DSQ EI low-resolution mass spectrometer (Thermo Fisher Scientific, Waltham, MA, USA) and a Thermo MAT95XP EI high-resolution mass spectrometer (Thermo Fisher Scientific). Preparative HPLC was performed using a Shimadzu LC-20AT HPLC pump equipped with an SPD-20A dual λ absorbance detector and a Shim-pack PRC-ODS HPLC column (250 × 20 mm) (Shimadzu Corporation, Nakagyo-ku, Kyoto, Japan).

### 3.2. Plant Material

The pericarps of *Garcinia mangostana* were collected from Guangzhou City, Guangdong Province, China, in June 2013. Voucher specimens are deposited in School of Chemistry and Chemical Engineering, Sun Yat-sen University.

### 3.3. Extraction and Isolation

The wet pericarps of *Garcinia mangostana* Linn (1 Kg) were extracted three times with 95% ethanol (2 L) at r.t. for 12 h. The extract was concentrated by low-temperature rotary evaporation. The ethanol extract (20 g) was chromatographed on a silica gel column with petroleum ether–EtOAc (100:0–0:100) and then EtOAc–MeOH (100:0–0:100) as the eluent to afford 15 fractions. Fr. 6 (1.08 g) was further purified by repeated preparative HPLC eluted with H_2_O–MeCN (60:40, v/v) to yield compounds **1** (6 mg), **3** (146 mg), **4** (168 mg) and **6** (45 mg). Fr. 7 (1.43 g) was further purified by HPLC with a gradient of H_2_O–MeCN (40:60 up to 0:100, v/v) to afford compounds **2** (12 mg), **5** (78 mg), and **7** (463 mg).

### 3.4. Spectral Data

*1,3,7-Trihydroxy-2-(3-methyl-2-butenyl)-8-(3-hydroxy-3-methylbutyl)xanthone* (**1**). Yellow solid. 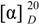: + 15.8 (*c* 0.03, MeOH); UV (MeOH) λ_max_ (ε) 207 nm (9,868), 247 nm (15,547), 287 nm (12,367), 350 nm (2,172); IR *v*_max_ 3229, 2964, 2927, 2855, 1642, 1609, 1582, 1485, 1460, 1373, 1319, 1306, 1281, 1231, 1209, 1182, 1161, 1125, 1100, 1083, 1054, 937, 904, 818, 797, 783 cm^−1^; ^1^H- and ^13^C-NMR data, see Table 1; LREIMS *m*/*z* 398, 380, 363, 337, 325, 309, 295, 281, 269, 257, 241, 213, 185, 167, 149, 130, 105, 91, 77, 69, 57; HREIMS *m*/*z* 398.1725 [M]^+^ (calcd for C_23_H_26_O_6_, 398.1724).

*1,3,8-Trihydroxy-2-(3-methyl-2-butenyl)-4-(3-hydroxy-3-methylbutanoyl)-xanthone* (**2**). Yellow amorphous solid. 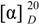: + 19.7 (*c* 0.014, MeOH); UV (MeOH) λ_max_ (ε) 210 nm (34,165), 242 nm (46,002), 264 nm (44,690), 312 nm (25,976), 370 nm (8,733); IR *v*_max_ 3466, 3176, 2956, 2926, 2854, 1638, 1600, 1578, 1500, 1464, 1428, 1374, 1356, 1339, 1292, 1238, 1224, 1197, 1167, 1129, 1104, 1170, 964, 847, 789, 779, 748, 723, 661 cm^−1^; ^1^H- and ^13^C-NMR data, see Table 1; LREIMS *m*/*z* 412, 394, 379, 354, 339, 323, 311, 299, 283, 271; HREIMS *m*/*z* 412.1517 [M]^+^ (calcd for C_23_H_24_O_7_, 412.1517).

*Garcinone C* (**3**). Yellow solid. ^13^C-NMR (DMSO-*d_6_*, 100 MHz) *δ*_C _: 181.2 (C), 161.6 (C), 159.6 (C), 153.9 (C), 152.1 (C), 151.7 (C), 140.4 (C), 129.9 (C), 129.7 (C), 122.3 (CH), 109.9 (C), 109.1 (C), 101.8 (C), 99.8 (CH), 91.8 (CH), 69.2 (C), 43.6 (CH_2_), 29.0 (2 × CH_3_), 25.4 (CH_3_), 21.8 (CH_2_), 20.9 (CH_2_), 17.6 (CH_3_). ^1^H-NMR (DMSO-*d_6_*, 400 MHz) *δ*_H _: 13.95 (s, 1H), 10.66 (s, 2H), 8.68 (s, 1H), 6.72 (s, 1H), 6.31 (s, 1H), 5.17 (t, *J* = 6.9 Hz, 1H), 4.23 (s, 1H), 3.31 (m, 2H), 3.20 (d, *J* = 6.9 Hz, 2H), 1.73 (s, 3H), 1.62 (s, 3H), 1.57 (m, 2H), 1.20 (s, 6H).

*Garcinone D* (**4**). Yellow solid. ^13^C-NMR (DMSO-*d_6_*, 100 MHz) *δ*_C _: 180.9 (C), 161.9 (C), 159.7 (C), 156.5 (C), 154.3 (C), 153.9 (C), 143.0 (C), 138.3 (C), 130.4 (C), 122.3 (CH), 109.9 (C), 109.4 (C), 101.7 (C), 101.4 (CH), 92.1 (CH), 69.1 (C), 60.4 (CH_3_), 44.8 (CH_2_), 29.0 (2 × CH_3_), 25.5 (CH_3_), 22.3 (CH_2_), 21.0 (CH_2_), 17.7 (CH_3_) . ^1^H-NMR (DMSO-*d_6_*, 400 MHz) *δ*_H_: 13.80 (s, 1H), 10.80 (s, 2H), 6.75 (s, 1H), 6.31 (s, 1H), 5.16 (t, *J* = 6.9 Hz, 1H), 4.17 (s, 1H), 3.74 (s, 3H), 3.27 (m, 2H), 3.19 (d, *J* = 6.9 Hz, 2H), 1.72 (s, 3H), 1.61 (s, 3H), 1.55 (m, 2H), 1.22 (s, 6H).

*Gartanin* (**5**). Yellow solid. ^13^C-NMR (DMSO-*d_6_*, 100 MHz) *δ*_C_: 183.9 (C), 161.4 (C), 156.8 (C), 152.0 (C), 151.6 (C), 143.5 (C), 137.2 (C), 130.8 (C), 130.7 (C), 123.2 (CH), 122.1 (CH), 122.0 (CH), 110.7 (C), 108.7 (CH), 107.0 (C), 106.8 (C), 101.1 (C), 25.6 (CH_3_), 25.5 (CH_3_), 21.4 (CH_2_), 21.2 (CH_2_), 17.8 (2 × CH_3_), 17.8 (CH_3_). ^1^H-NMR (DMSO-*d_6_*, 400 MHz) *δ*_H _: 12.15 (s, 1H), 11.16 (s, 1H), 9.56 (s, 2H), 7.21 (d, *J =* 8.7 Hz, 1H), 6.57 (d, *J* *=* 8.7 Hz, 1H), 5.20 (t, *J* = 6.9 Hz, 1H), 5.13 (t, *J* = 6.9 Hz, 1H), 3.53 (d, *J* = 6.9 Hz, 2H), 3.31 (d, *J* = 6.9 Hz, 2H), 1.81 (s, 3H), 1.74 (s, 3H), 1.63 (s, 6H).

*Xanthone I* (**6**). Yellow solid. ^13^C-NMR (DMSO-*d_6_*, 100 MHz) *δ*_C _: 181.8 (C), 159.8 (C), 157.8 (C), 156.2 (C), 155.6 (C), 154.6 (C), 142.6 (C), 137.0 (C), 132.2 (C), 127.2 (CH), 123.2 (CH), 115.7 (CH), 112.1 (C), 104.0(C), 103.7 (C), 101.8 (CH), 94.2 (CH), 78.0 (C), 62.2 (CH_3_), 28.5 (2 × CH_3_), 28.5 (CH_3_), 26.7 (CH_3_), 26.1 (CH_3_), 18.5 (CH_3_). ^1^H-NMR (DMSO-*d_6_*, 400 MHz) *δ*_H_: 13.66 (s, 1H), 6.78 (s, 1H), 6.70 (d, *J* = 10 Hz, 1H), 6.21 (s, 1H), 5.55 (d, *J* = 10 Hz), 5.25 (t, *J* = 5.8 Hz, 1H), 4.06 (d, *J* = 5.8 Hz, 2H), 3.80 (s, 3H), 1.83 (s, 3H), 1.70 (s, 3H), 1.47 (s, 6H).

*γ-Mangostin* (**7**). Yellow solid. ^13^C-NMR (DMSO-*d_6_*, 100 MHz) *δ*_C _: 181.3 (C), 161.8 (C), 159.6 (C), 154.0 (C), 152.3 (C), 151.7 (C), 140.7 (C), 130.1 (C), 129.9 (C), 127.5 (C), 123.6 (CH), 122.5 (CH), 109.9 (C), 109.2 (C), 101.9 (C), 100.0 (CH), 92.0 (CH), 25.7 (CH_3_), 25.5 (CH_3_), 25.4 (CH_2_), 21.0 (CH_2_), 18.1 (CH_3_), 17.7 (CH_3_). ^1^H-NMR (DMSO-*d_6_*, 400 MHz) *δ*_H_: 13.85 (s, 1H), 10.55 (s, 2H), 8.85 (s, 1H), 6.73 (s, 1H), 6.31 (s, 1H), 5.19 (t, *J* = 6.3 Hz, 2H), 5.17 (t, *J* = 6.3 Hz, 2H), 4.03 (d, *J* = 6.3 Hz, 2H), 3.21 (d, *J* = 6.9 Hz, 2H), 1.77 (s, 3H), 1.73 (s, 3H), 1.62 (s, 6H).

### 3.5. Cytotoxicity Assay

The *in vitro* cytotoxicities of **1**–**7** was determined by means of the colorimetric 3-(4,5-dimethylthiazol-2-yl)-2,5-diphenyl-2*H*-tetrazolium bromide (MTT) assay. The tested human cancer cell lines were seeded in 96-well plates at a density of 3 × 10^7^ cells/L, and the compounds were added at various concentrations (0.125–50 mg/L). After 48 h, MTT was added to the culture medium at a final concentration of 0.5 mg/mL, and the plates were incubated for 4 h at 37 °C. The supernatant was removed. The formazan crystals were dissolved in DMSO (150 µL) with gentle shaking at r.t. The absorbance at 570 nm was recorded with a microplate reader (Bio-Rad, Hercules, CA, USA), and the data were analyzed with the SPSS 13.0 software package. Hirsutanol A, a potent anticancer agent isolated from marine fungal metabolites, was used as a positive control and its cytotoxic activities against the tested cancer cell lines were showed in Table 2 [14].

## 4. Conclusions

The pericarps of *Garcinia mangostana* contain numerous xanthones with chemical diversity. The hydroxyl and prenyl groups are the common function groups in the xanthones, however, the variations in the number and position, as well as the diverse prenyl side chain derivatives, including hydroxyl, double bond, and cyclic compounds, make up a large xanthone library. Hopefully, a systematic exploration of the constituents of the pericarps may afford additional xanthones. Compound **2** has a ketone group on the side chain, which is unusual in naturally-occurring xanthones. Compounds **1**–**7** showed potent cytotoxic activities. These results provide additional support for the medicinal applications of *Garcinia mangostana*.

## Supplementary Materials

Supplementary materials can be accessed at: http://www.mdpi.com/1420-3049/19/2/1820/s1.
