# Supplementary material for: Cytotoxic Prenylated Xanthones from the Pericarps of Garcinia mangostana
Source: Molecules. 2014 Feb 6;19(2):1820–7. doi: 10.3390/molecules19021820 (PMC6271135; doi:10.3390/molecules19021820)

Supplementary Materials

Table of Contents

Figure S1: HREIMS of compound **1** S2
Figure S2: ^{1}H-NMR (400 MHz, acetone-d_6) spectrum of compound **1** S3
Figure S3: ^{13}C-NMR (100 MHz, acetone-d_6) spectrum of compound **1** S4
Figure S4: gHMQC of compound **1** S5
Figure S5: ^{1}H–^{1}H gCOSY of compound **1** S6
Figure S6: gHMBC of compound **1** S7
Figure S7: HREIMS of compound **2** S8
Figure S8: ^{1}H-NMR (400 MHz, acetone-d_6) spectrum of compound **2** S9
Figure S9: ^{13}C-NMR (100 MHz, acetone-d_6) spectrum of compound **2** S10
Figure S10: gHMQC of compound **2** S11
Figure S11: ^{1}H–^{1}H gCOSY of compound **2** S12
Figure S12: gHMBC of compound **2** S13

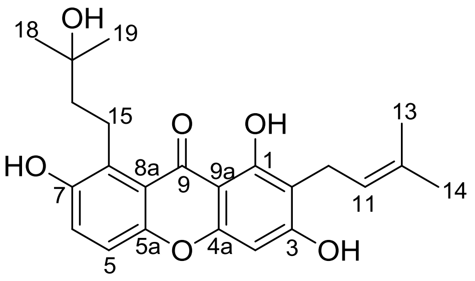

1,3,7-trihydroxy-2-(3-methyl-2-butenyl)-8-(3-hydroxy-3-methylbutyl)-xanthone (**1**)

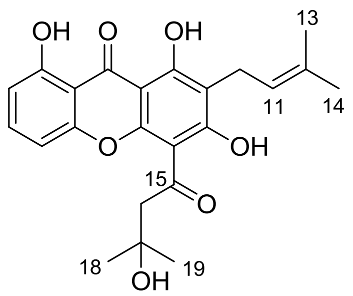

1,3,8-trihydroxy-2-(3-methyl-2-butenyl)-4-(3-hydroxy-3-methylbutanoyl)-xanthone (**2**)

Figure S1. HREIMS of compound **1**.

SPECTRUM - MS
File : D:\DATA-HR\13\092605-sz-c3.RAW
Full ms [390.500 - 408.500] - Range: 398.100 - 398.300
Scan No. 1 of 23
Scan #: 1
RT: 0.04
Data points: 1

Mass	Relative Intensity	Theoretical Mass	Delta [ppm]	Delta [mmu]	RDB	Composition
398.1725	9.1	398.1724	0.3	0.1	11.0	C_{23} H_{26} O_6

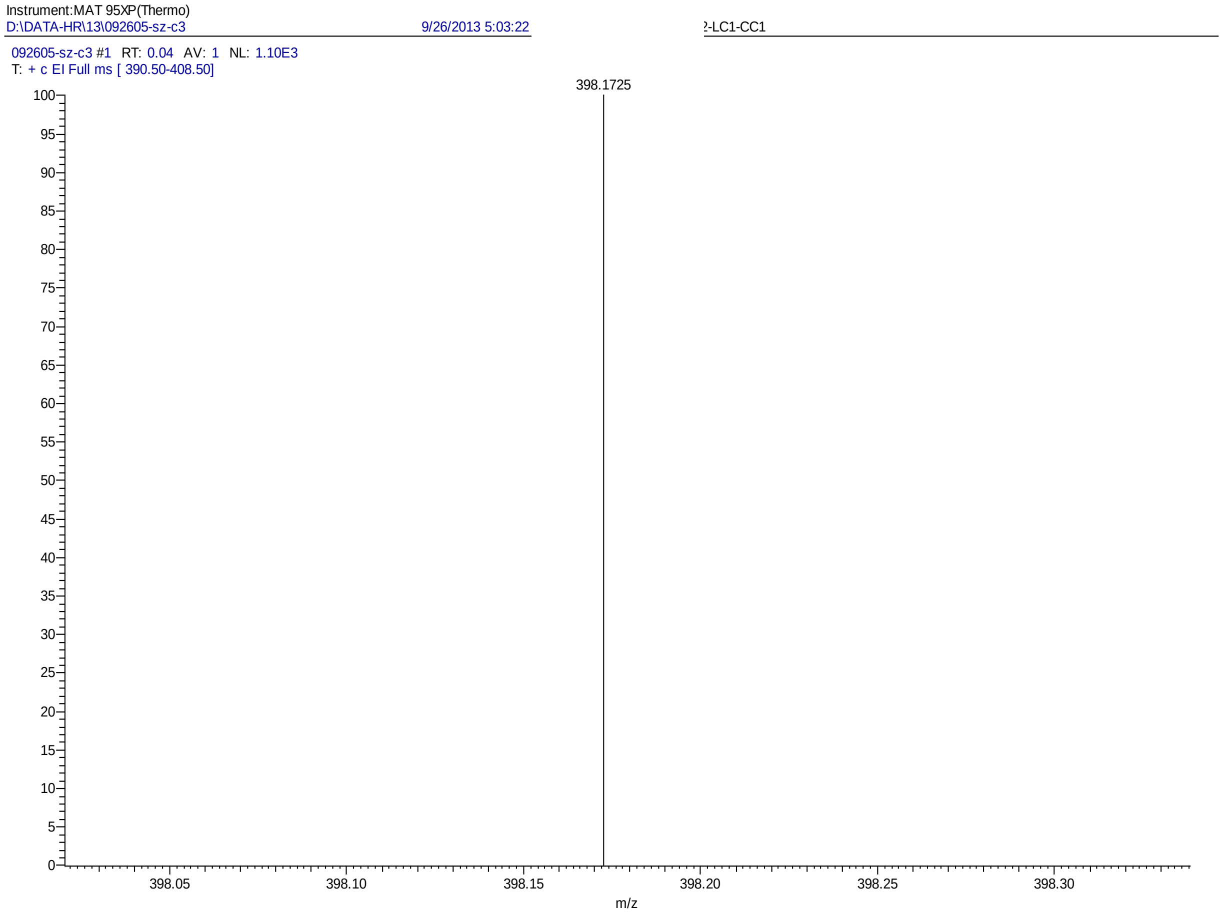

Figure S2. ^{1}H-NMR (400 MHz, acetone-d_6) spectrum of compound **1**.

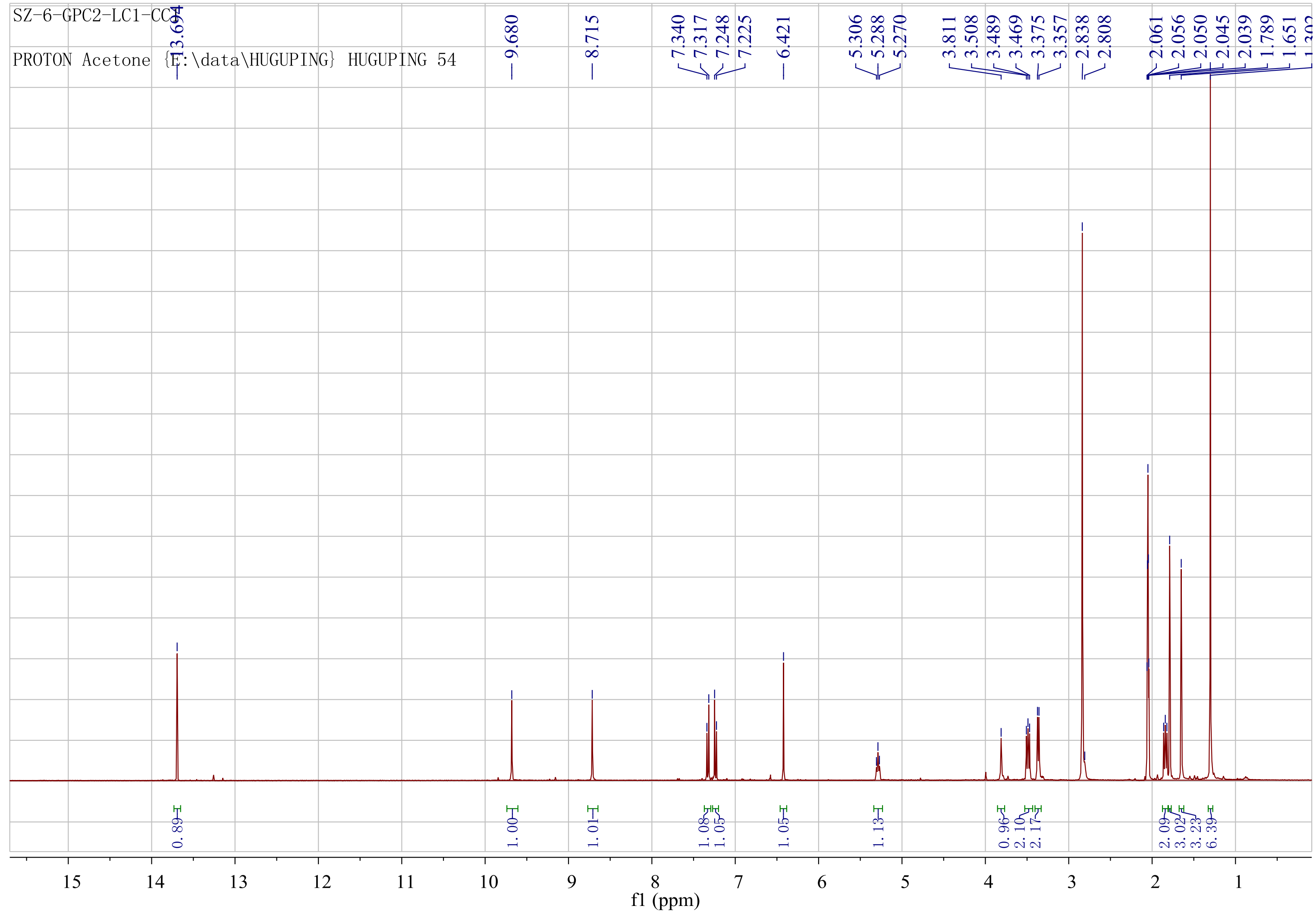

Figure S3. ^{13}C-NMR (100 MHz, acetone-d_6) spectrum of compound **1**.

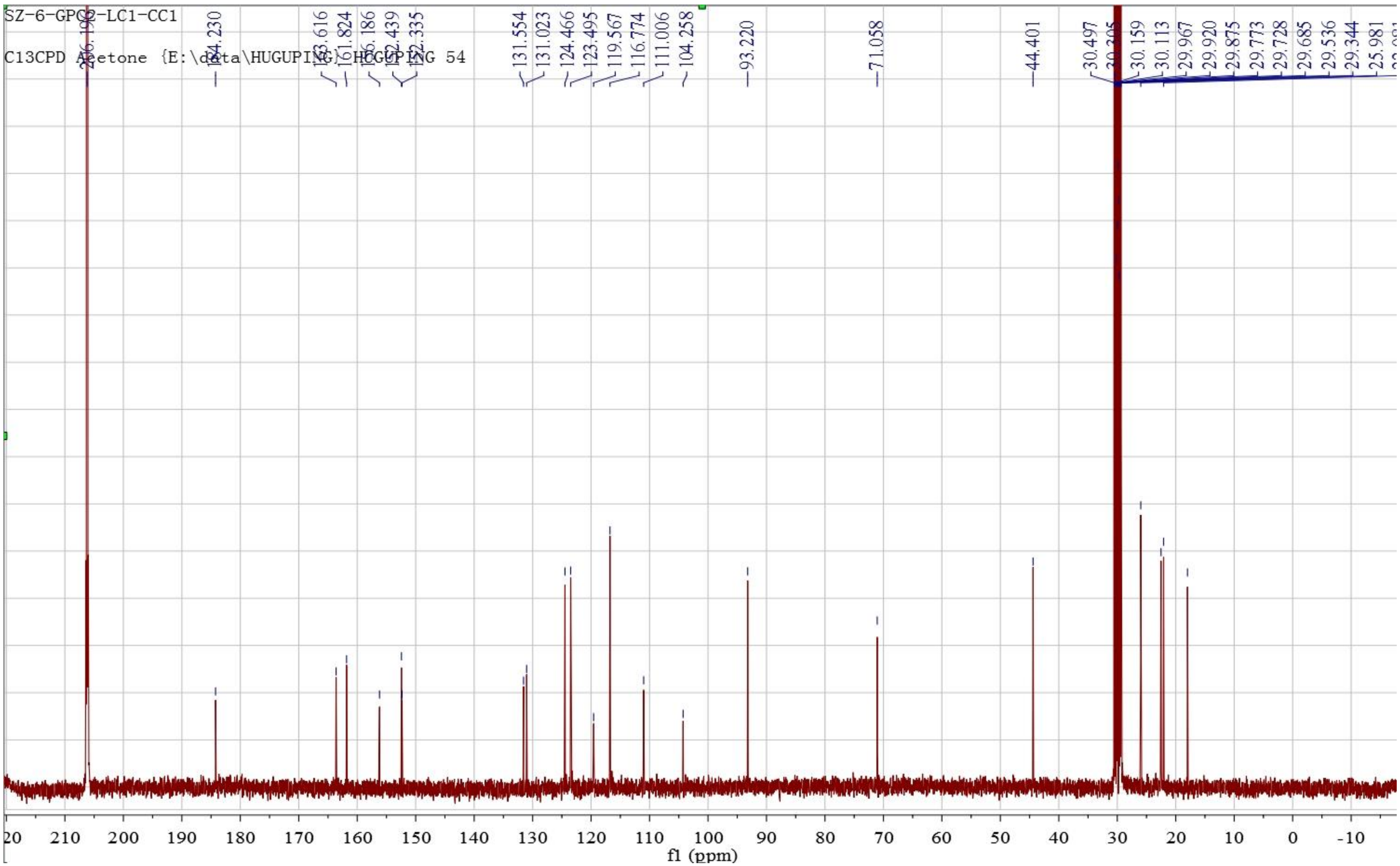

Figure S4. gHMQC of compound **1**.

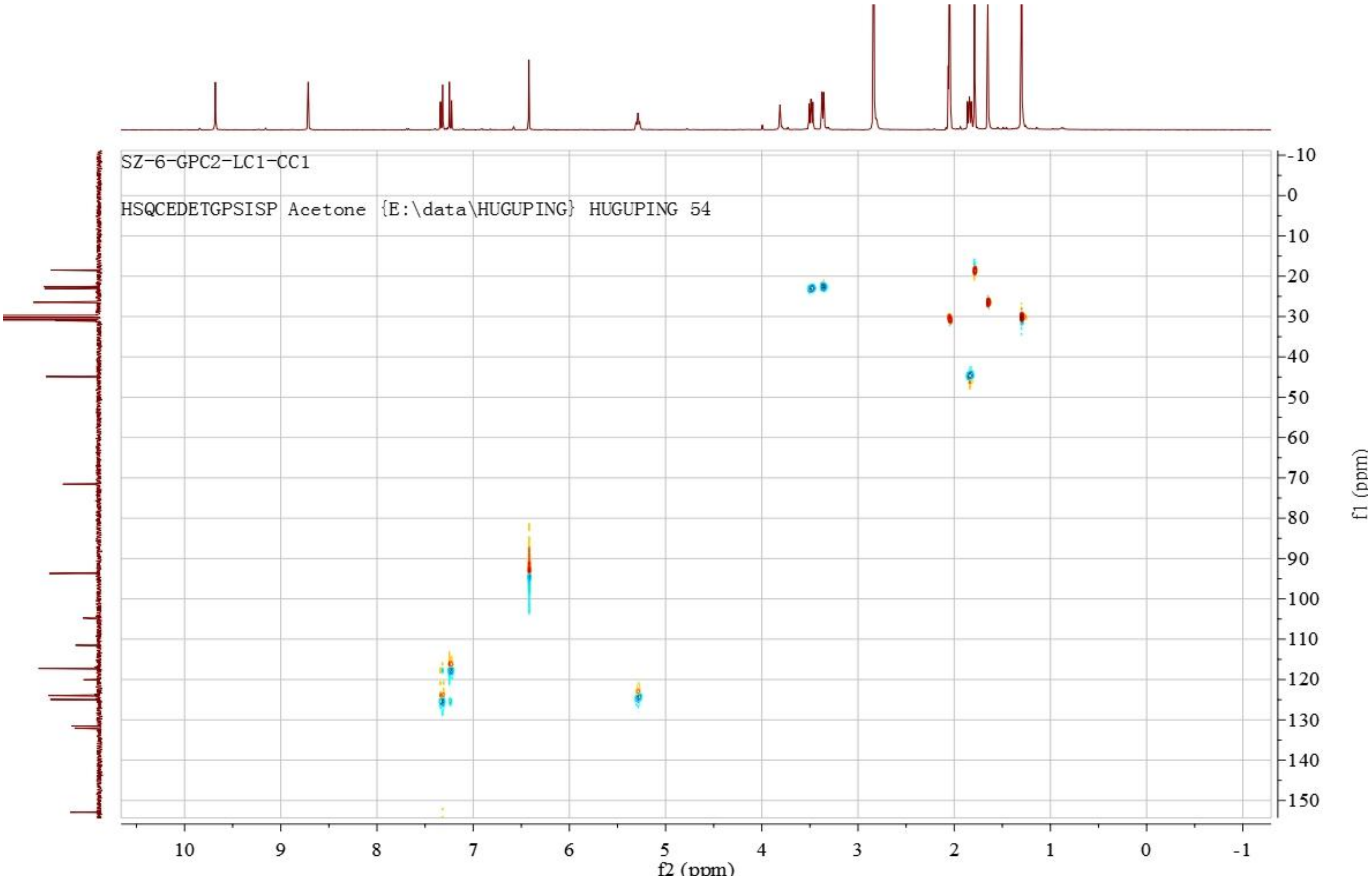

Figure S5. ^{1}H–^{1}H gCOSY of compound **1**.

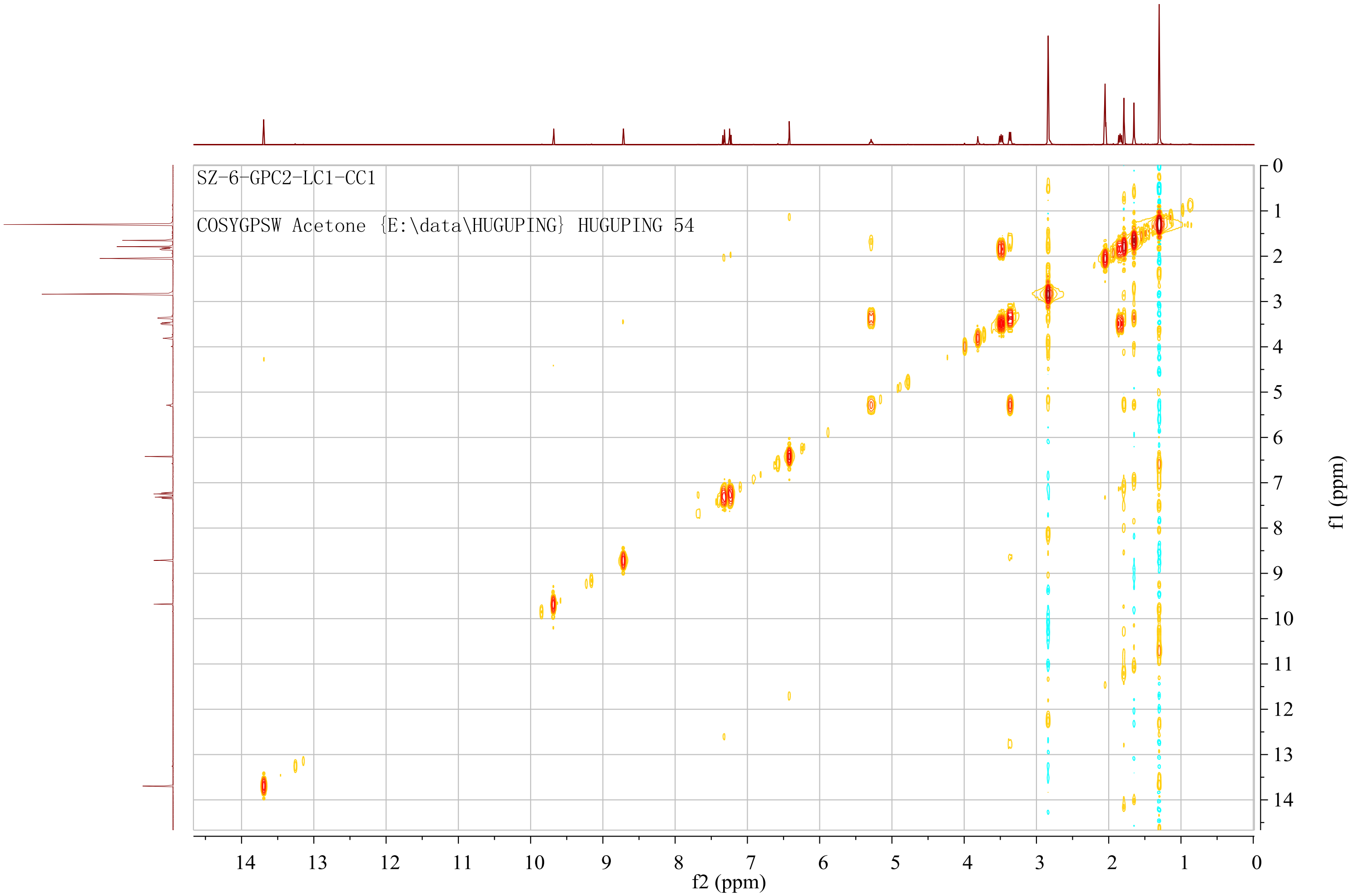

Figure S6. gHMBC of compound **1**.

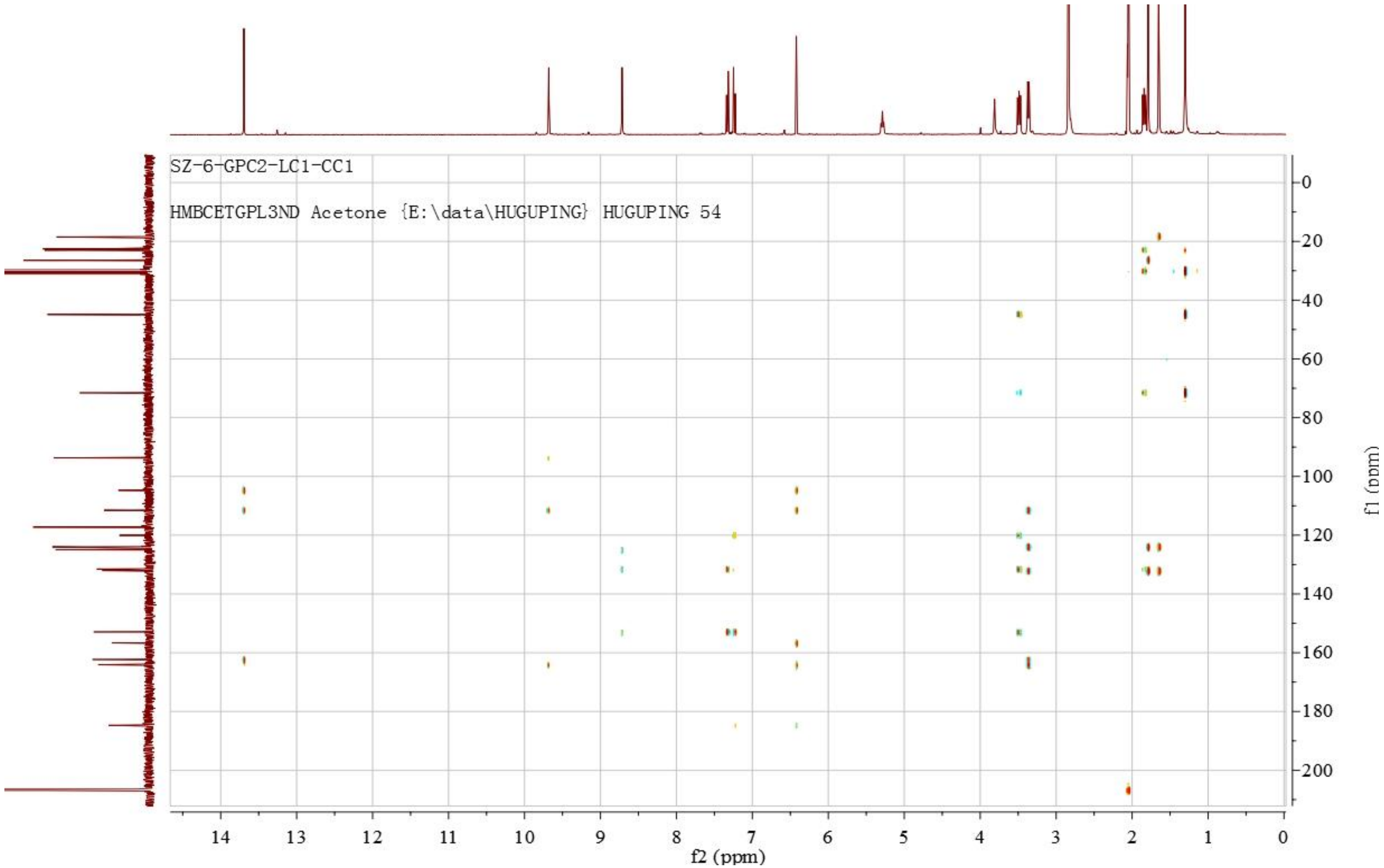

Figure S7. HREIMS of compound **2**.

SPECTRUM - MS
File : D:\DATA-HR\13\060902-sz-2-c1.RAW
Full ms [400.500 - 421.500] - Range: 412.000 - 412.390
Scan No. 12 of 21
Scan #: 12
RT: 0.45
Data points: 1

Mass	Relative Intensity	Theoretical Mass	Delta [ppm]	Delta [mmu]	RDB	Composition
412.1517	38.8	412.1517	0.1	0.0	12.0	C_{23} H_{24} O_7

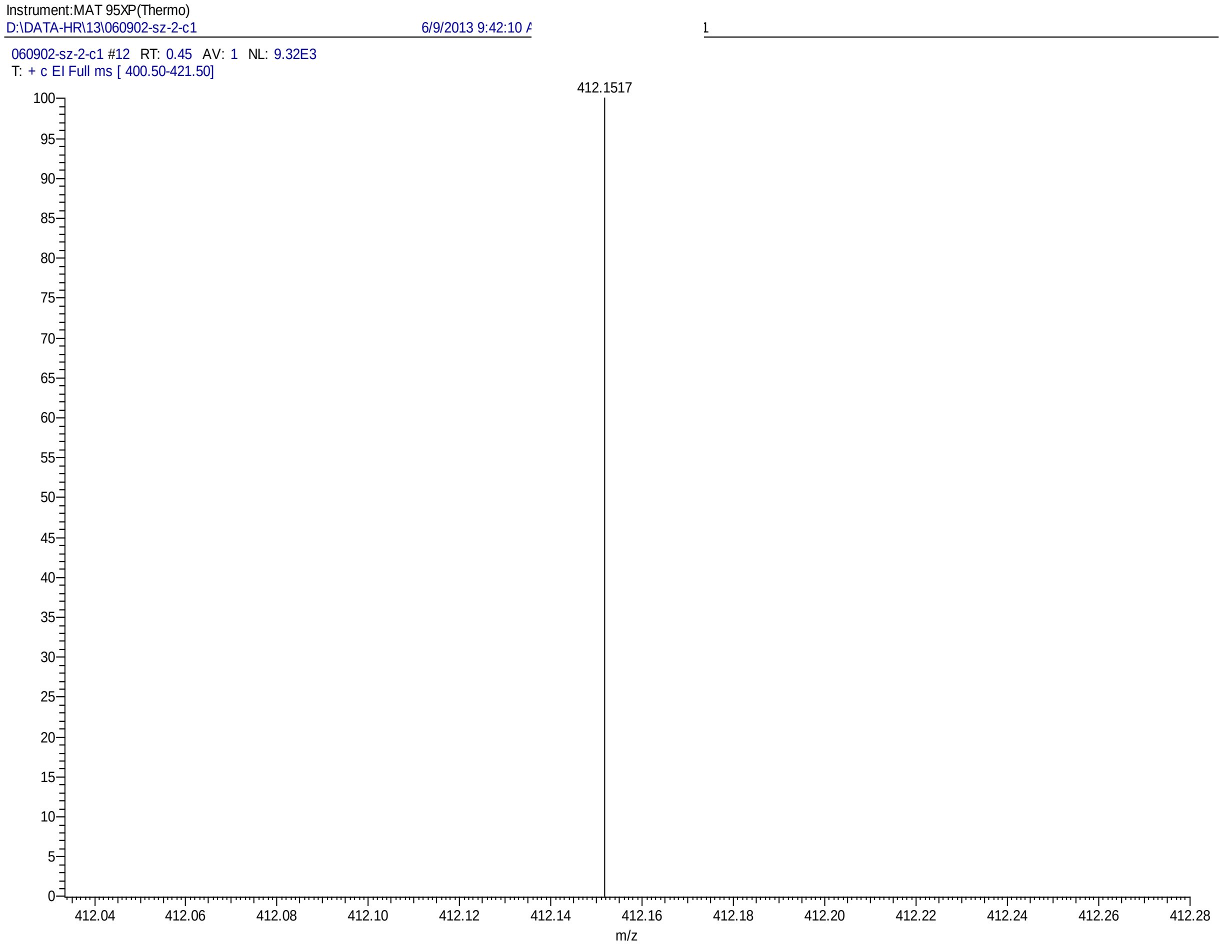

Figure S8. ^{1}H-NMR (400 MHz, acetone-d_6) spectrum of compound **2**.

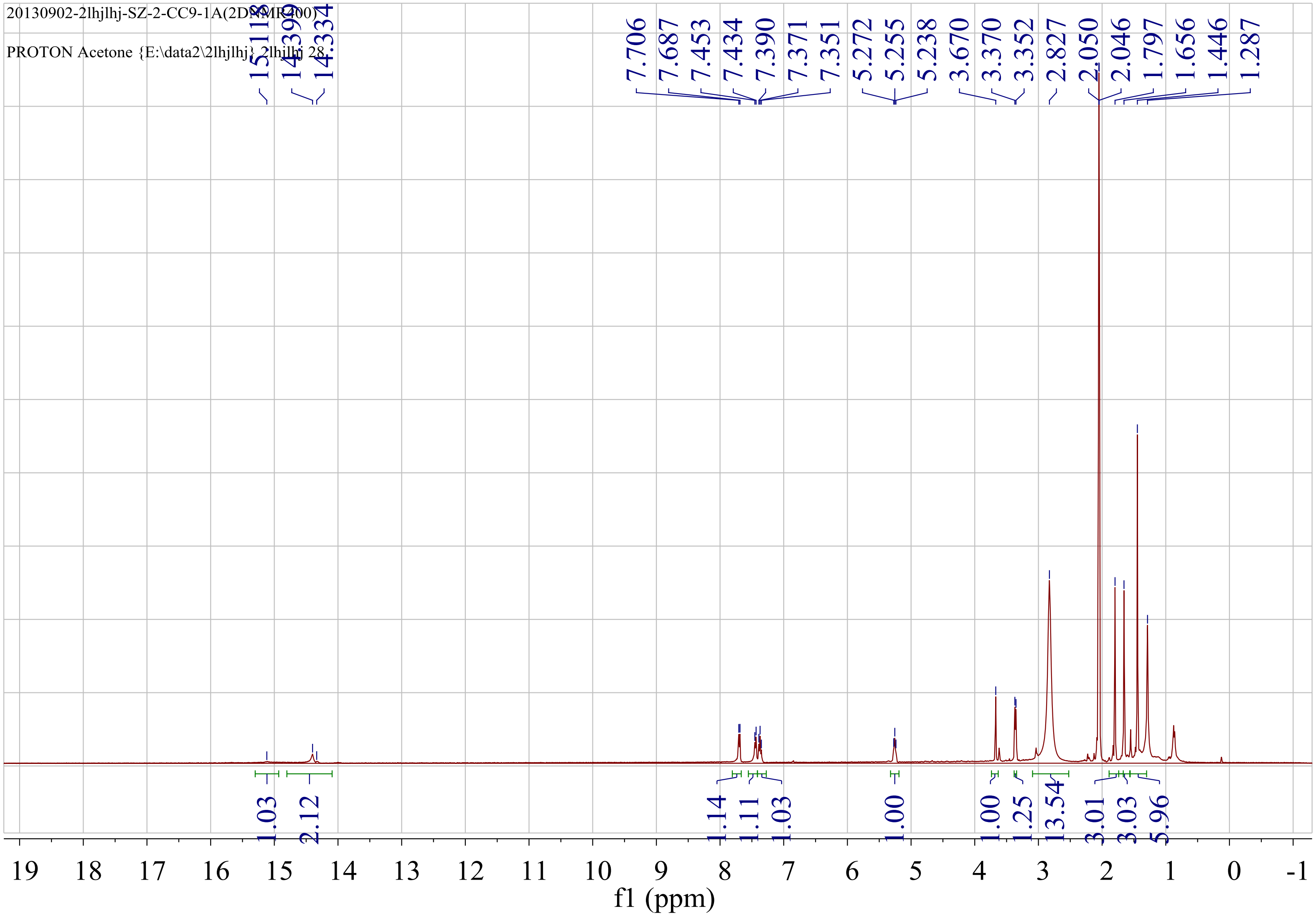

Figure S9. ^{13}C-NMR (100 MHz, acetone-d_6) spectrum of compound **2**.

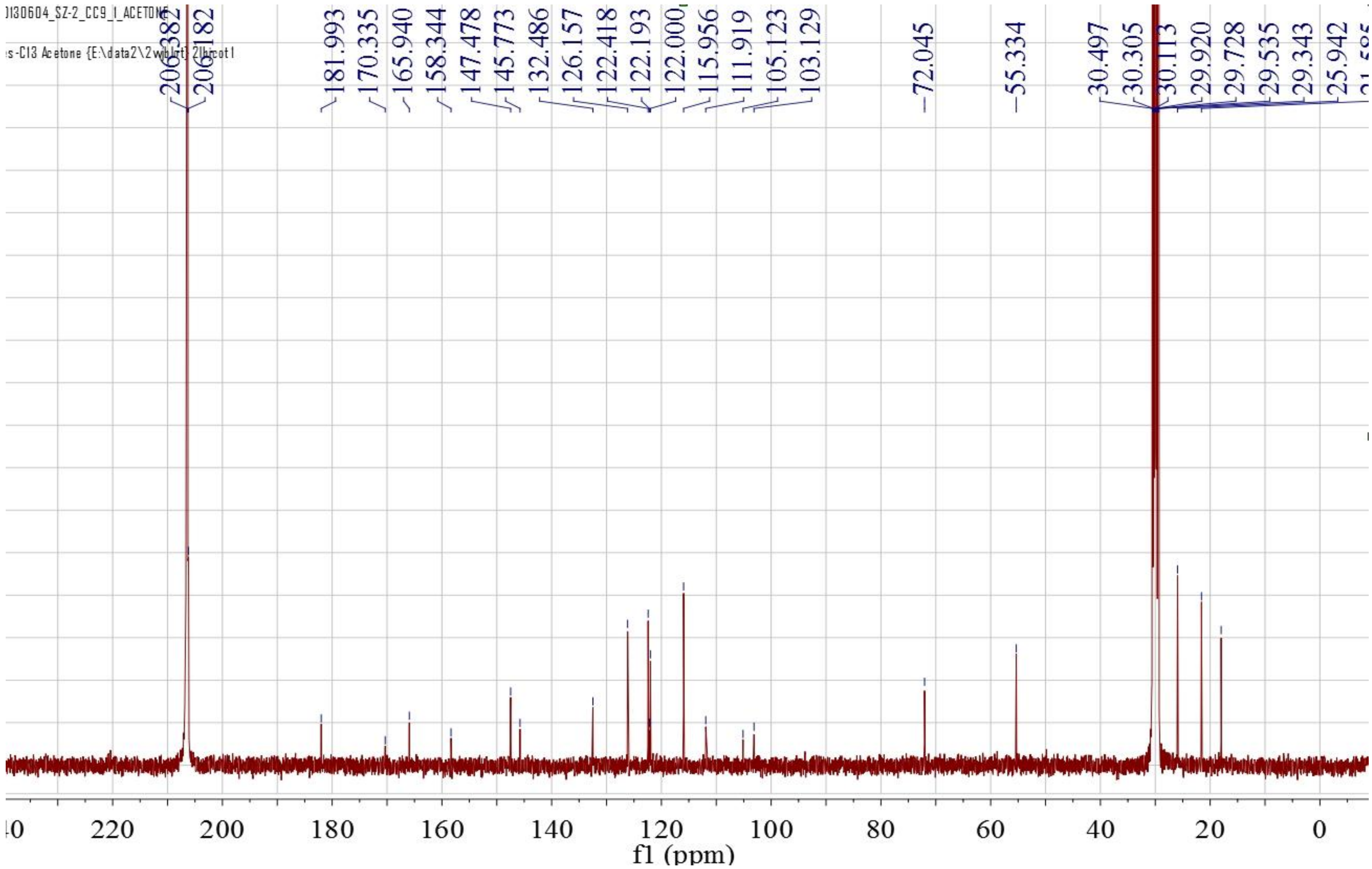

Figure S10. gHMQC of compound **2**.

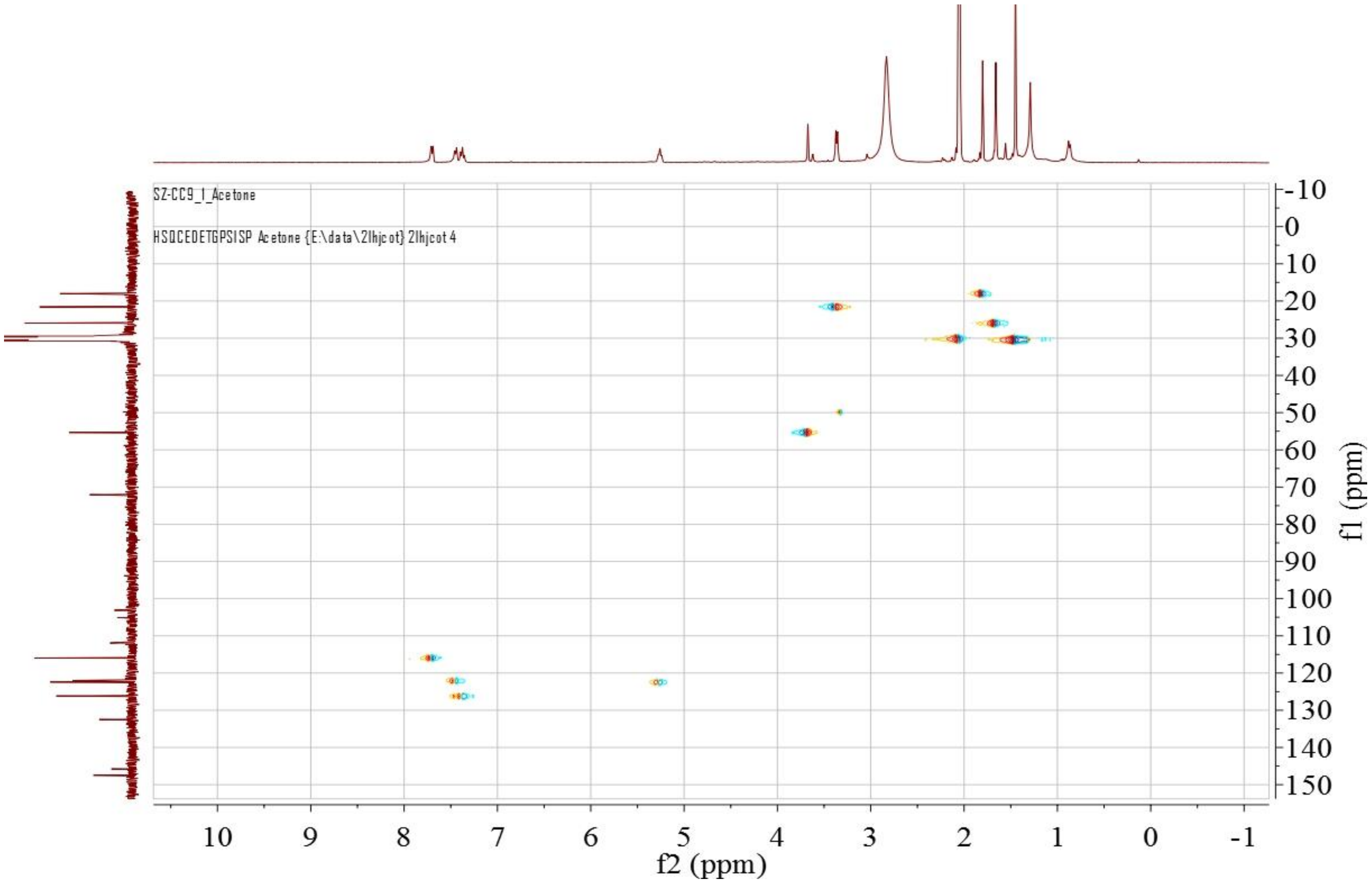

Figure S11. $^{1}H–^{1}H$ gCOSY of compound **2**.

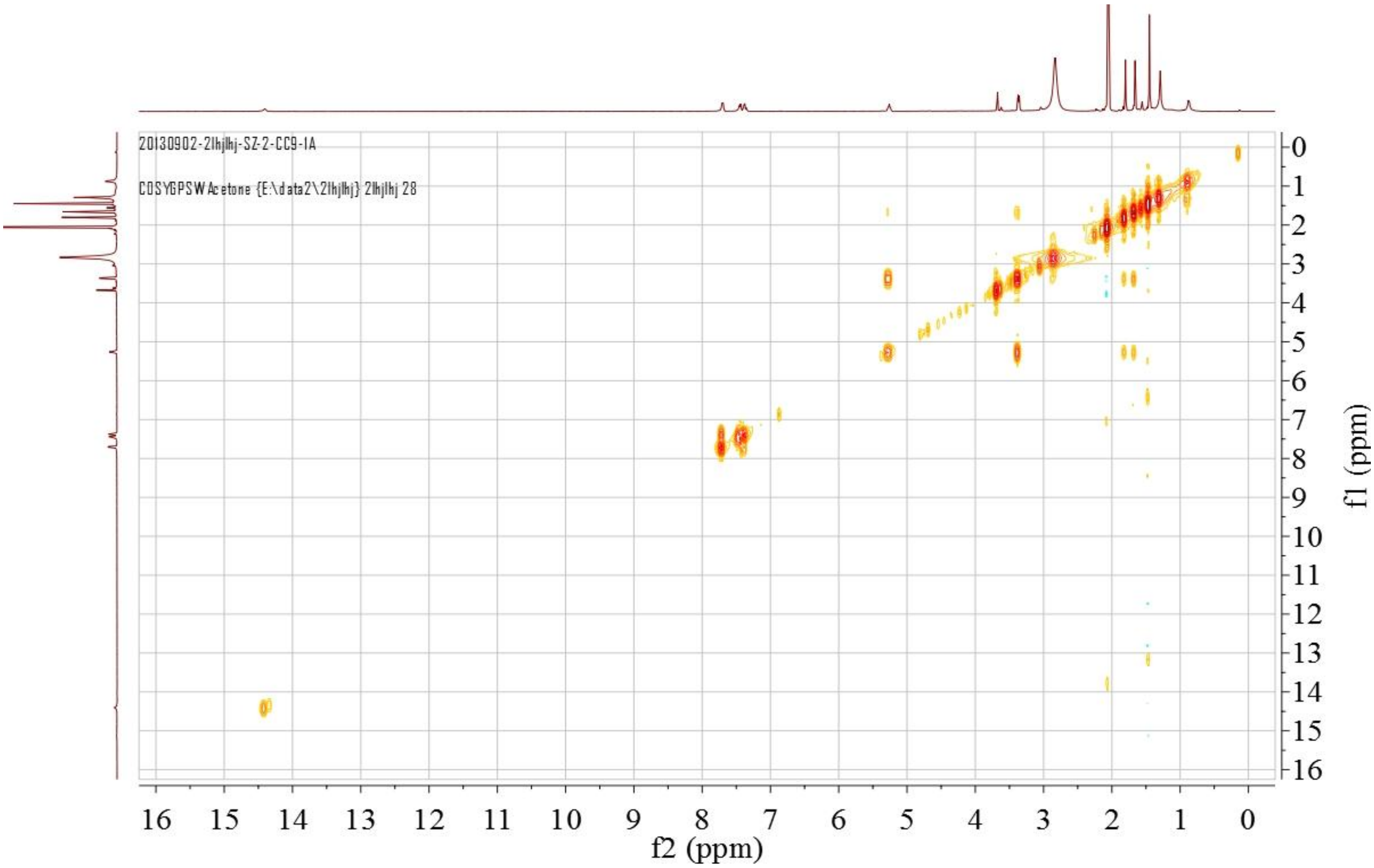

Figure S12. gHMBC of compound **2**.

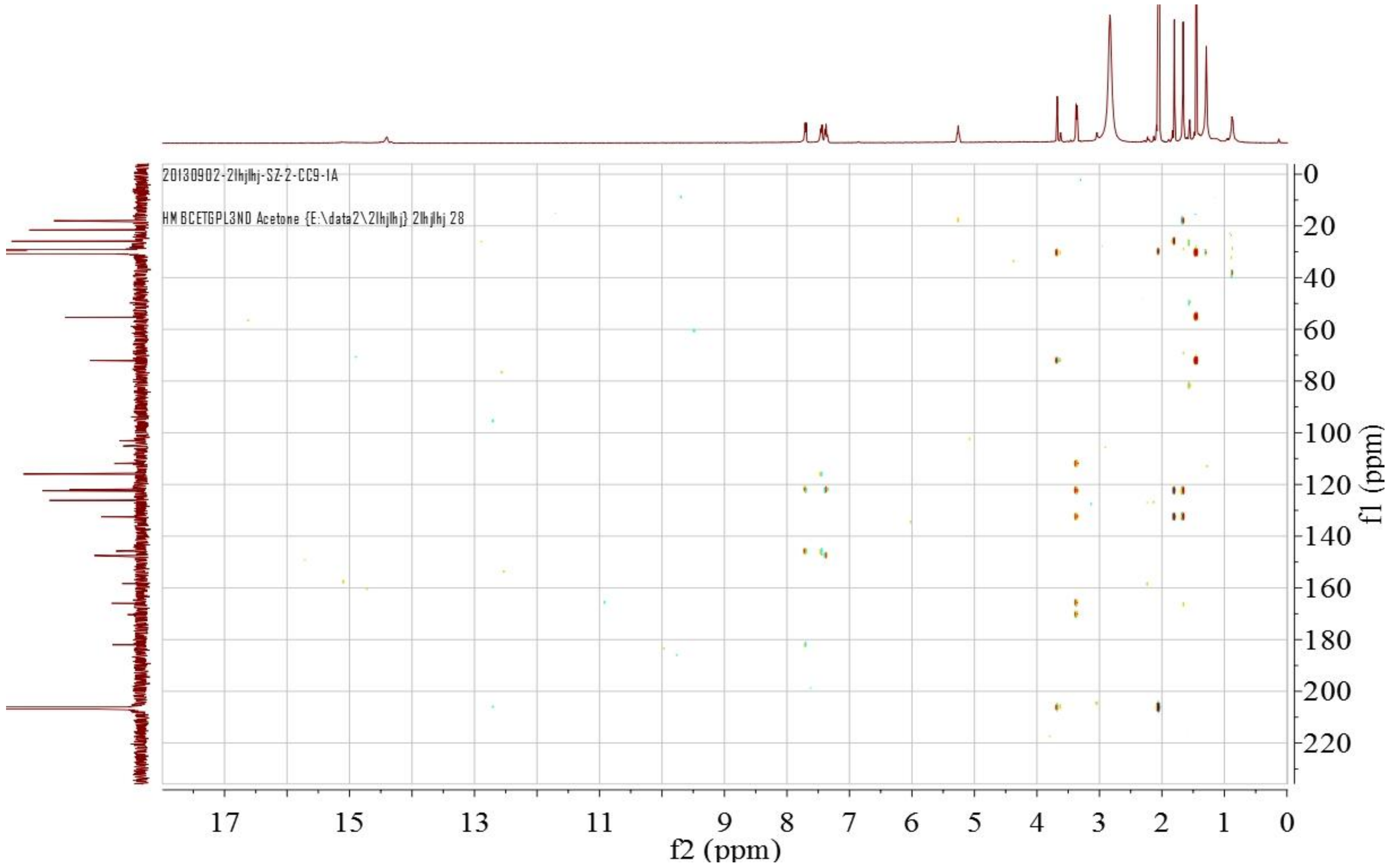

Supplement: Supplementary file 1 [file molecules-19-01820-s001.pdf]
